# Supplementary material for: Targeting APE1 endonuclease activity impairs metastasis and enhances genotoxic therapy response in pancreatic cancer
Source: Res Sq. 2025 Dec 16:rs.3.rs-8197122. Preprint. [Version 1] doi: 10.21203/rs.3.rs-8197122/v1 (PMC12776480; doi:10.21203/rs.3.rs-8197122/v1)
Supplement: 1 [file NIHPPRS8197122V1-supplement-1.pdf]

Supplemental Fig S1: APE1 endonuclease loss sensitizes cells to alkylating agent-induced necrosis.

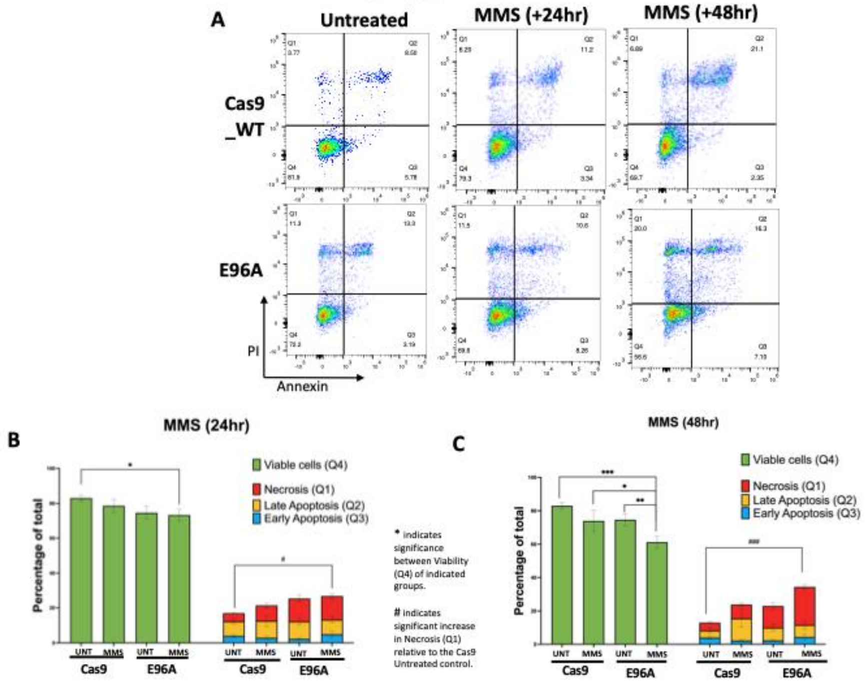

Supplemental Table S1: Generation and Viability Outcomes of APE1 Knock-In Mutant PDAC Cell Lines

Mutation/ Edit	Function Impacted	Clones Screened	Number Of Positive Clones Established	Viability Of Edited Pools	Notes On Isolation Outcome
**APEX1: C65A**	Redox	~190	4	High (>90% Indel/KI)	Screening halted after establishing sufficient biological replicates.
**APEX1: E96A**	Repair	~95	3	High (>90% Indel/KI)	Screening halted after establishing sufficient biological replicates.
**APEX1: D210N**	Repair	>285	0	Low	Genotype verified in bulk pool but failed to survive clonal isolation (presumed lethal).
**APEX1: C65A-E96A**	Redox-Repair	~95	1	Low	Significant attrition observed. Only one clone survived expansion.

Supplemental Fig S2: Expression of pol B complex proteins is unchanged in E96A mutant cell lines

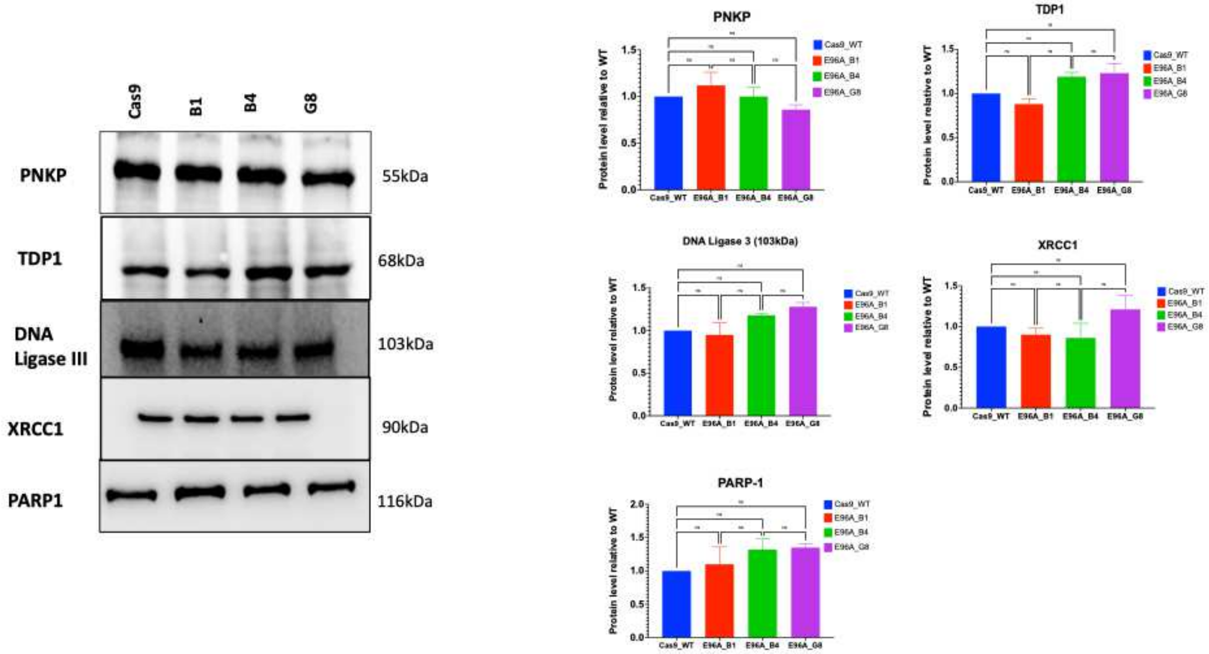

Supplemental Fig 3: Knockdown of potential backup proteins did not sensitize E96A cell lines to alkylating treatment

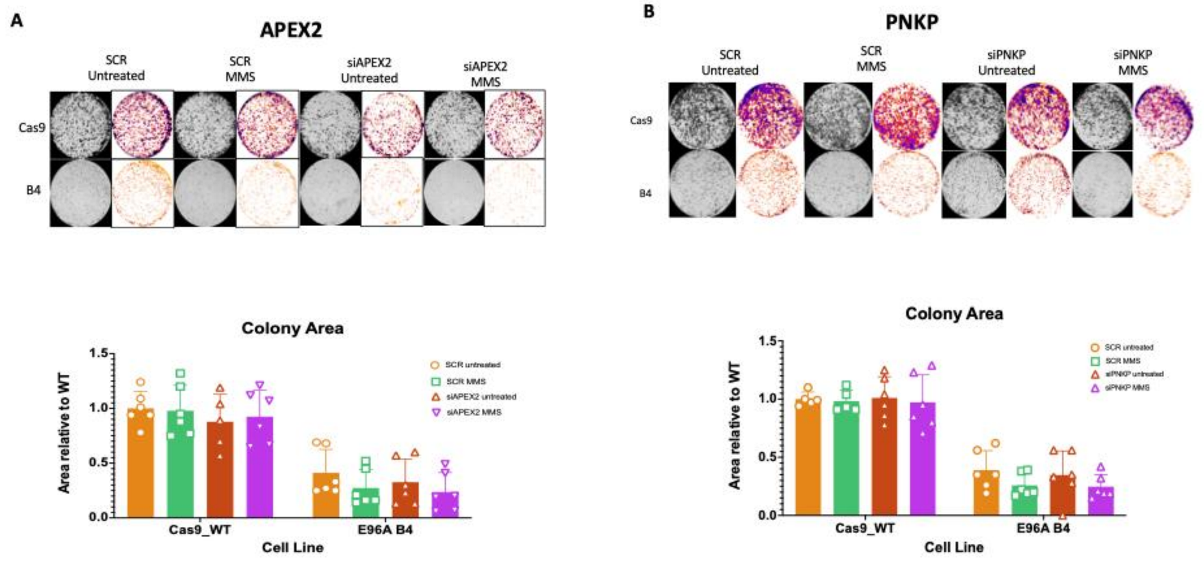

Supplemental Figure 1: APE1 endonuclease loss sensitizes cells to alkylating agent-induced necrosis

(A) Representative flow cytometry plots of Annexin V/Propidium Iodide (PI) staining in Cas9 control and E96A cells following exposure to 900µM Methyl methanesulfonate (MMS). Cells were analyzed at 24 h and 48 h post-treatment.

(B-C) Quantification of cell fate distributions at (B) 24 h and (C) 48 h. Stacked bars display the proportion of viable (Q4), early apoptotic (Q3), late apoptotic (Q2), and necrotic (Q1) cells. Data represent mean ± SEM (n=3). Statistical significance was determined by two-way ANOVA with Tukey's multiple comparisons. Asterisks indicate significant differences in cell viability (Q4) between genotypes (**$p < 0.01$). Hashes (#) indicate a significant increase in necrosis (Q1) relative to the untreated Cas9 control (#$p < 0.05$, ###$p < 0.001$).

Supplemental Figure 2: Expression of BER complex proteins is unchanged in E96A mutants

(Left) Representative Western blot analysis of key Base Excision Repair (BER) and Single-Strand Break Repair (SSBR) proteins (PNKP, TDP1, DNA Ligase III, XRCC1, and PARP1) in Cas9 control and E96A mutant cell lines.

(Right) Densitometric quantification of protein abundance normalized to Vinculin and expressed relative to the Cas9 control. Data represent mean ± SEM (n=3). Statistical significance was determined by one-way ANOVA (ns, not significant).

Supplemental Figure 3: Knockdown of potential backup enzymes does not restore genotoxic sensitivity in E96A cells

(A) Effect of APEX2 knockdown. (Top) Representative images of methylene blue-stained colonies and corresponding binary segmentation masks for Cas9 control and E96A (clone B4) cells transfected with Scramble (SCR) or APEX2-targeting siRNA, followed by exposure to MMS (225µM, 30 min pulse). (Bottom) Quantification of colony area normalized to the untreated SCR control for each cell line.

(B) Effect of PNKP knockdown. (Top) Representative images and segmentation masks for SCR and siPNKP transfected cells. (Bottom) Quantification of colony area.
Data represent mean ± SEM (n=3). No significant differences were observed between SCR and target-specific knockdown conditions within treatment groups (one-way ANOVA; $p > 0.05$).

Supplemental Table S1: Generation and Viability Outcomes of APE1 Knock-In Mutant PDAC Cell Lines

Supplemental Table S2: Guide RNA and donor sequences used for E96A CRISPR knock-in

Supplemental Table S3: List of antibodies utilized

Supplemental Table S4: List of primers and siRNA utilized
